# Ancient DNA Analyses Reveal Contrasting Phylogeographic Patterns amongst Kiwi (*Apteryx* spp.) and a Recently Extinct Lineage of Spotted Kiwi

**DOI:** 10.1371/journal.pone.0042384

**Published:** 2012-08-02

**Authors:** Lara D. Shepherd, Trevor H. Worthy, Alan J. D. Tennyson, R. Paul Scofield, Kristina M. Ramstad, David M. Lambert

**Affiliations:** 1 Allan Wilson Centre, Massey University, Auckland, New Zealand; 2 Institute of Fundamental Sciences, Massey University, Palmerston North, New Zealand; 3 Museum of New Zealand Te Papa Tongarewa, Wellington, New Zealand; 4 School of Biological, Earth and Environmental Sciences, University of New South Wales, Sydney, Australia; 5 Canterbury Museum, Christchurch, New Zealand; 6 Allan Wilson Centre, School of Biological Sciences, Victoria University of Wellington, Wellington, New Zealand; 7 Griffith School of Environment and School of Biomolecular and Physical Sciences, Griffith University, Nathan, Australia; University of Illinois at Urbana-Champaign, United States of America

## Abstract

The little spotted kiwi (*Apteryx owenii*) is a flightless ratite formerly found throughout New Zealand but now greatly reduced in distribution. Previous phylogeographic studies of the related brown kiwi (*A. mantelli*, *A. rowi* and *A. australis*), with which little spotted kiwi was once sympatric, revealed extremely high levels of genetic structuring, with mitochondrial DNA haplotypes often restricted to populations. We surveyed genetic variation throughout the present and pre-human range of little spotted kiwi by obtaining mitochondrial DNA sequences from contemporary and ancient samples. Little spotted kiwi and great spotted kiwi (*A. haastii*) formed a monophyletic clade sister to brown kiwi. Ancient samples of little spotted kiwi from the northern North Island, where it is now extinct, formed a lineage that was distinct from remaining little spotted kiwi and great spotted kiwi lineages, potentially indicating unrecognized taxonomic diversity. Overall, little spotted kiwi exhibited much lower levels of genetic diversity and structuring than brown kiwi, particularly through the South Island. Our results also indicate that little spotted kiwi (or at least hybrids involving this species) survived on the South Island mainland until more recently than previously thought.

## Introduction

Kiwi (*Apteryx* spp.) are flightless ratites endemic to New Zealand. Currently five species are recognized in two morphological groups: spotted kiwi, comprising little spotted kiwi (*A*. *owenii*) and great spotted kiwi (*A*. *haastii*), and brown kiwi, comprising North Island brown kiwi (*A*. *mantelli*), rowi (*A*. *rowi*) and tokoeka (*A*. *australis*).

In this study we examine phylogeographic structuring in little spotted kiwi and compare our results to those obtained previously for brown kiwi, which exhibit one of the most striking phylogeographic patterns observed in any bird worldwide [1]. Mitochondrial DNA (mtDNA) sequences from the reduced, disjunct modern populations of the three brown kiwi species revealed an extremely high level of genetic structuring, with almost every population possessing private mtDNA haplotypes [2], [3], a pattern more akin to that often seen in mammals rather than birds [1], [2]. Analysis of ancient brown kiwi samples from regions where they are now extinct indicated that this structuring, with even higher levels of genetic variation, also existed in the past and was not therefore the result of human-mediated extinction [4].

Great spotted kiwi (*A. haastii*) occur in the northwest of the South Island and have a distribution that has apparently not diminished in response to human arrival to the same extent as the other kiwi species [4]. However, great spotted kiwi numbers continue to decrease and this species is considered nationally vulnerable [5]. In contrast to great spotted kiwi, the distribution of subfossil (i.e. late Pleistocene to Holocene) bones of little spotted kiwi (*A. owenii*), which are significantly smaller than the bones of other kiwi, indicate that prior to human arrival this species occurred throughout the North and South Islands [6]. Since European settlement, only two live little spotted kiwi have been collected in the North Island, both in the 19th century [7], [8], and there were several additional sightings [9], [10]. The extinction of little spotted kiwi in the South Island was poorly documented, with the misidentification of great spotted kiwi likely causing confusion. Despite reports that this species was still common on the West Coast in the 1970 s [11], there have been few confirmed records in the last 70 years. Since the discovery of a specimen on the South Island mainland in 1938 [12], there have been only two verified reports of recently living South Island little spotted kiwi (a feather, and leg bones), both from Fiordland [13]. However, there are a number of small spotted kiwi specimens held in museums that were collected more recently whose identities have been debated [14]–[16] (note that in [15] the registration number of the cited specimen on page 307 should be NMNZ OR.23036). These specimens were collected from within or adjacent to the recorded range of great spotted kiwi and some authors considered them to be juvenile great spotted kiwi. Determining the identity of these specimens would clarify the timing of extinction of mainland little spotted kiwi.

**Figure 1:**
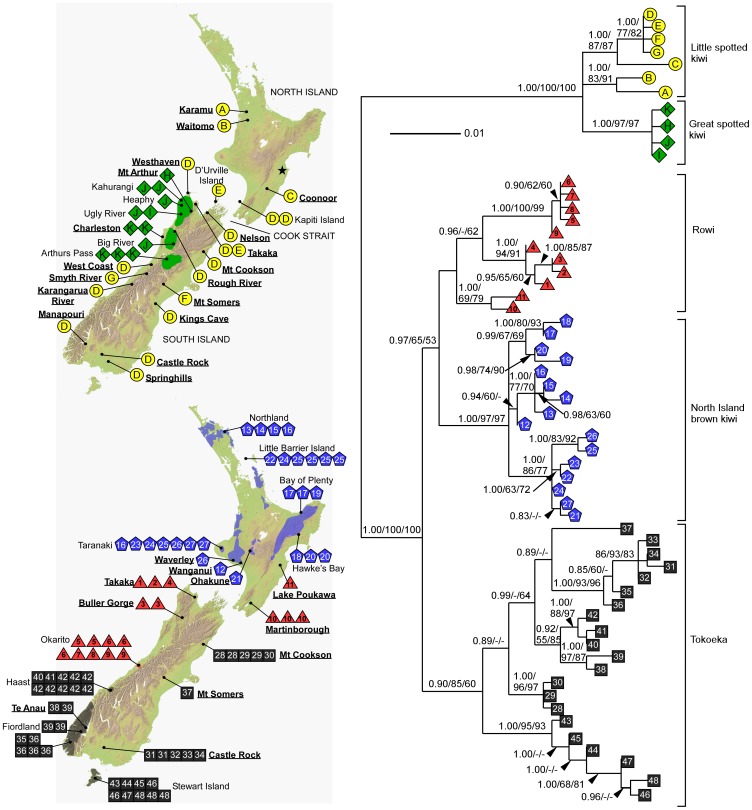
A. Distribution of spotted kiwi haplotypes. The current distribution of great spotted kiwi is depicted in green. Little spotted kiwi presently (2012) occur on several predator-free offshore islands and a wildlife sanctuary on the mainland (not shown); all derive from transfers from Kapiti Island or D’Urville Island (little spotted kiwi have been removed from the latter). Little spotted kiwi haplotypes are represented by circles; great spotted kiwi samples are indicated by diamonds. Only the approximate position of the sample from ‘West Coast, South Island’ (museum number AV25141) is indicated on the map because of the imprecision of its recorded locality. A star indicates the position of the sample (AV17079) from which a partial DNA sequence identical to the control region of haplotype A was obtained. B. Distribution of brown kiwi haplotypes. The current distribution of North Island brown kiwi is shown in blue and their haplotypes are shown as pentagons. The distribution of rowi is shown in red and their haplotypes as triangles. The distribution of tokoeka is shown in grey and tokoeka haplotypes are shown as squares. In both maps the location names of ancient samples are shown in bold and underlined. C. Midpoint-rooted Bayesian phylogeny of the expanded sample set which included all spotted and brown kiwi samples and all loci (missing loci were coded as missing data). Numbers above the branches represent posterior probabilities (PP), MP and ML bootstrap (BS) values, respectively. Only PP>0.70 and BS>50% are shown.

By the 1980 s little spotted kiwi only survived in two populations, both on offshore islands (Kapiti Island and D’Urville Island; Figure 1). The origin of the Kapiti Island population has been debated with suggestions that it is a natural remnant population or derives from a recent unrecorded translocation. A number of bird species have been introduced to Kapiti Island since it was declared a sanctuary in 1897, including brown kiwi, and it has been suggested that the little spotted kiwi population derives from such a translocation [12], [17], [18]. However, historical records of kiwi translocations to Kapiti Island are vague with regard to species [19]. Little spotted kiwi declined to such low numbers on D’Urville Island that the few remaining birds were removed. Little spotted kiwi have also been translocated from Kapiti Island to a number of other islands and a mainland sanctuary [20]. Little spotted kiwi presently number around 1500 individuals with numbers increasing [21]. They are conservation dependent [5] and are classified as ‘near threatened’ on the IUCN Red List [22].

Genetic research is being undertaken on the modern populations of little spotted kiwi, [23]. However, there has been no survey of the pre-decline genetic variation across the former distribution of this taxon. Historical accounts [24], [25] and fossil bones [26]–[29] show that little spotted and brown kiwi were previously sympatric in many areas. Therefore, ancient little spotted kiwi from across New Zealand might be expected to have been influenced by the same historical factors as brown kiwi and thus potentially exhibit a similar high level of phylogeographic structuring and cryptic taxonomic diversity.

In this study our primary aim is to examine the genetic structure of little spotted kiwi across its former range using mtDNA sequences from subfossil bones and museum skins. Our secondary aims are to investigate the origin of the Kapiti Island little spotted kiwi population and to determine the species identification of three possible little spotted kiwi specimens collected from the South Island during the last 60 years.

## Materials and Methods

Thirty-four ancient specimens identified as little spotted kiwi by morphology were obtained for this study (Tables S1 and S2). Permission for accessing and sampling these specimens was approved by Alan Tennyson (Museum of New Zealand Te Papa Tongarewa), Paul Scofield (Canterbury Museum), Neville Hudson (Auckland University) and Kevan Wilde (Waitomo Caves Discovery Centre). These samples were selected to cover the past range of little spotted kiwi (although the paucity of samples from the North Island precluded more extensive sampling in this region; Figure 1). A further three specimens collected from the South Island, whose identifications have been debated, were included in our sampling (OR.1174, collected 1952; OR.23036 and OR.23043 both collected 1978, Table S1). Modern great spotted kiwi (n = 9) and little spotted kiwi (n = 3) blood samples (Table S3) were also included.

Ancient DNA extractions were performed in a dedicated ancient DNA laboratory (Massey University, Albany campus, Auckland). This laboratory was regularly UV-irradiated and physically isolated from where PCR products were handled and modern DNA extractions performed. Negative controls were used throughout the extraction and PCR amplification processes. For one little spotted kiwi sample (WO255, Table S1) DNA was extracted from a whole vertebra. From another (NMNZ OR.23036, Table S1), DNA was extracted from a partial rib. The remaining little spotted kiwi bones (femora) were sampled by either removing a section using a Dremel grinder (NMNZ samples) or by drilling (samples from CM and AU). The surface layer of bones that were sampled with a Dremel grinder was removed by sanding with a Dremel wheel that was changed between each sample. Segments of 1 cm×0.3 cm were then cut from the centre of each bone and finely powdered in a coffee grinder that was cleaned between each sample with ethanol and regularly irradiated with UV light. The remaining bone samples were drilled using a 3 mm drill bit and the shavings collected. The drill bit was cleaned with bleach between each sample.

Museum skins were sampled by removing a sliver of approximately 3 mm^2^ of kiwi footpad tissue from the underside of the foot with a clean razor blade. For one museum skin (NMNZ OR.22007, Table S1) a single feather was removed in addition to a toe pad for verification purposes (see below). The basal 2 mm of the feather shaft was used for DNA extraction. Ancient bone samples were decalcified and a phenol-chloroform extraction performed [4]. DNA was extracted from skin, feather and the modern blood samples by proteinase digestion followed by phenol-chloroform extraction [30].

Two samples were independently extracted for verification purposes in the ancient DNA laboratory at the University of Auckland. The small size of little spotted kiwi bones prevented them from being sampled a second time without substantially damaging the integrity of the bone. Instead verification was achieved by analysis of two samples, a feather and a toe pad, taken from one museum skin specimen (NMNZ OR.22007, Table S1). Femora from two different individuals from Earl Grey Cave were also sampled (NMNZ S.27784.1 and NMNZ S.27784.2, Table S1).

Primers (Table S4) were designed to amplify 190 bp of domain 1 of the mtDNA control region, a 257 bp amplicon including portions of ATPase 6 and ATPase 8, and 471 bp of cytochrome *b* in two overlapping fragments and one non-contiguous fragment. PCR amplification and sequencing were performed for each primer pair using the protocol described in [4]. All amplicons were sequenced in both directions from independent PCR amplifications.

DNA sequences were edited using Sequencher™ 3.1.1 (Gene Codes Corporation) and aligned manually. For some analyses the spotted kiwi sequences obtained here were manually aligned to published sequences of brown kiwi and ancient great spotted kiwi [2]–[4].

A neighbornet was constructed using SplitsTree 4.8 [31] to examine the support and conflict for each split (bipartition in the data) in the spotted kiwi sequences. ATPase sequences were not available for the published ancient brown kiwi and ancient great spotted kiwi samples. Therefore, phylogenetic tree building was performed on two datasets: (1) the reduced sample set was restricted to the samples where all 871 bp of sequence was available (2) the expanded sample set included all samples and all loci (with missing loci included as missing data). Maximum parsimony (MP) phylogenies were constructed with PAUP* version 4.0b10 [32] using a heuristic search algorithm with 10 (expanded sample set) or 100 (reduced sample set) random addition sequences and tree bisection-reconnection (TBR) branch swapping. Maximum likelihood (ML) phylogenies were constructed using PhyML [33] implemented in Geneious ver 5.3.4 [34], using the GTR+I+G model for the expanded sample set and the GTR+G model for the reduced sample set (determined with ML optimized base trees for each model and the corrected Akaike Information Criterion (AICc) in jModeltest 0.1.1 [35]). For both MP and ML analyses nodal support was assessed by 1000 bootstrap replicates.

A Bayesian inference approach was also used to estimate phylogenetic relationships (MrBayes 3.1.2 [36]), with substitution parameters unlinked between the three loci, nst = 6, rates = invgamma and the default priors. Two concurrent analyses were run, each with four Markov chains of 10 million generations. The chains were sampled every 1000 generations, and the first 50% of these samples were discarded as ‘burn-in’. At this point, the standard deviation of split frequencies was less than 0.01, indicating convergence to a stationary distribution had been achieved. Convergence was also monitored with Tracer 1.4.1 [37] by confirming that effective sample size values were >200.

The number of haplotypes, haplotype (*h*) diversity and nucleotide (π) diversity was determined for each kiwi species using ARLEQUIN version 3.5.1.2 [38]. Only the 661 bp of DNA sequence that was obtained for all 115 kiwi samples was used.

## Results

Seventeen of the thirty-seven samples of ancient and historic little spotted kiwi yielded full-length DNA sequences of 871 bp (Table S1). Partial sequences were obtained from a further three samples (Table S2); these were not used for further analyses. The samples extracted and amplified at the ancient DNA laboratory at the University of Auckland had identical sequences to the corresponding samples processed at Massey University.

Thirty-seven variable sites, of which twenty were parsimony informative, were present in the alignment of 32 ancient and modern spotted kiwi sequences (Table 1). Three haplotypes, defined by two variable sites, were detected from the nine modern great spotted kiwi sequences. The 20 little spotted kiwi sequences exhibited twenty-seven variable sites defining seven haplotypes. Most of the variation in little spotted kiwi occurred among the three North Island samples, with each possessing a different haplotype (haplotypes A, B, C; Figure 1). In contrast, the majority of the ancient samples of little spotted kiwi from the South Island (11 of 14 samples) had the same haplotype (haplotype D). The modern little spotted kiwi samples from Kapiti Island also had haplotype D, as did the three spotted kiwi specimens from the South Island whose identifications have been debated.

**Table 1:** Variable sites defining spotted kiwi haplotypes, with their sequence alignment position indicated.

	Control region										Cytochrome *b*																	ATPase									
	57	76	81	82	83	86	142	146	177	181	59	92	137	140	170	260	281	290	314	320	347	377	390	392	402	419	434	4	45	51	71	72	150	170	185	203	207
**Great spotted kiwi**																																					
I	C	T	A	C	G	A	C	A	T	A	C	C	T	C	C	G	T	C	C	A	T	G	A	C	C	T	A	C	A	C	C	T	G	G	G	A	C
J	.	.	.	.	.	.	.	.	C	.	.	.	.	.	.	.	.	.	.	.	.	.	.	.	.	.	.	.	.	.	.	.	.	.	.	.	.
K	.	.	.	.	.	.	.	.	.	.	.	T	.	.	.	.	.	.	.	.	.	.	.	.	.	.	.	.	.	.	.	.	.	.	.	.	.
H	.	C	.	.	.	.	.	.	.	.	.	.	.	.	.	.	.	.	.	.	.	.	.	.	.	.	.	-	-	-	-	-	-	-	-	-	-
**Little spotted kiwi**																																					
A	.	.	.	.	.	.	T	G	.	.	.	.	.	T	.	.	.	T	T	G	C	T	.	.	A	C	G	.	.	T	.	C	.	A	A	G	T
B	.	.	.	.	.	G	.	.	.	.	.	.	.	.	.	A	.	T	T	.	C	T	.	.	A	C	.	.	.	T	.	C	.	.	A	G	T
C	T	.	.	T	.	.	.	.	.	G	T	.	C	.	.	.	C	T	T	.	.	T	G	.	.	.	.	A	G	T	T	.	.	A	A	G	T
D	.	.	G	.	.	.	.	.	.	G	.	.	.	.	T	.	.	T	T	.	.	T	G	T	.	.	.	A	G	T	.	.	.	A	A	G	T
E	.	.	G	T	.	.	.	.	.	G	.	.	.	.	T	.	.	T	T	.	.	T	G	T	.	.	.	A	G	T	.	.	.	A	A	G	T
F	.	.	G	.	.	.	.	.	.	G	.	.	.	.	T	.	.	T	T	.	.	T	G	T	.	.	.	A	G	T	.	.	.	A	A	G	T
G	.	.	G	.	A	.	.	.	.	G	.	.	.	.	T	.	.	T	T	.	.	T	G	T	.	.	.	A	G	T	.	.	A	A	A	G	T

The haplotype and nucleotide diversities (Table 2) of the two spotted kiwi species were significantly lower than those of the brown kiwi species (*t*-tests, P<0.0002 for each comparison except nucleotide diversity of little spotted kiwi versus North Island brown kiwi, P = 0.022).

**Table 2:** Genetic diversity measures for the 661 bp mitochondrial DNA sequence from the five kiwi species.

Taxon	No. of samples	No. of haplotypes	Haplotype diversity (*h*) ±SD	Nucleotide diversity (π) ±SD
Little spotted kiwi	20	6	0.516±0.132	0.005±0.003
Great spotted kiwi	12	4	0.742±0.084	0.002±0.001
North Island brown kiwi	26	15	0.920±0.041	0.008±0.005
Rowi	18	9	0.895±0.048	0.014±0.007
Tokoeka	39	21	0.941±0.023	0.020±0.010

**Figure 2:**
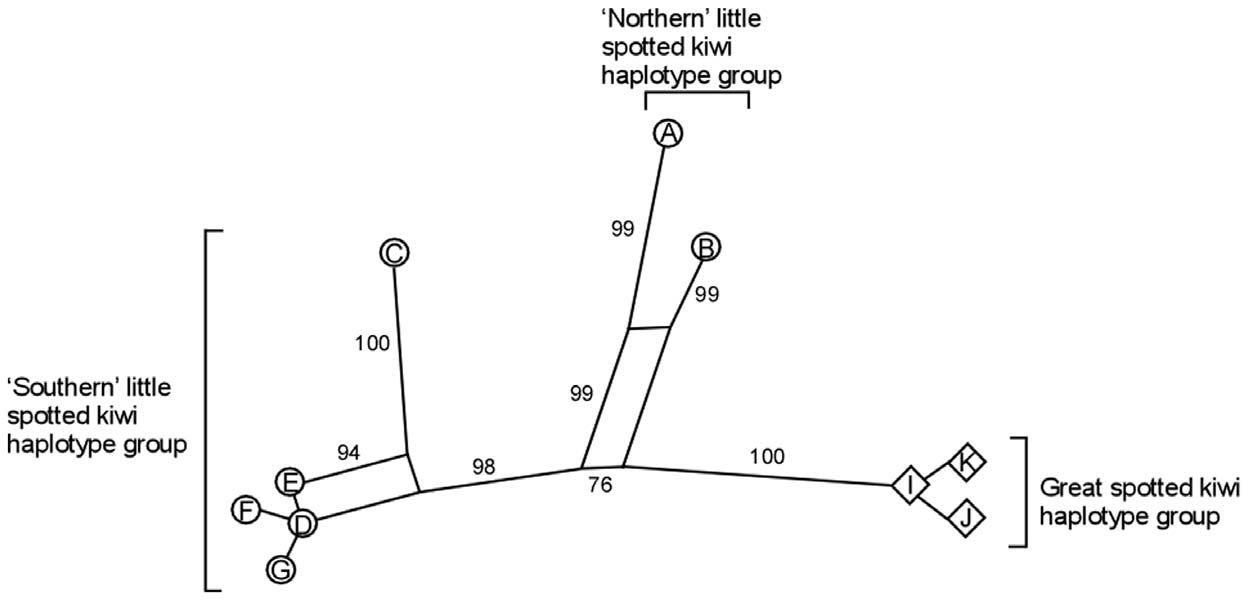
Neighbornet of spotted kiwi samples for which cytochrome *b*, control region and ATPase sequence was obtained.

The NeighborNet of the spotted kiwi sequences indicated limited conflict, represented by boxes, in the data (Figure 2). The MP, ML and MrBayes analyses of both datasets gave largely concordant results and revealed three well-supported lineages of spotted kiwi (Figure 1 and S1). Great spotted kiwi formed a distinct lineage and sequences from samples identified by morphology as little spotted kiwi formed the remaining two lineages. These two little spotted kiwi lineages were spatially separated, with haplotypes A and B from the northern North Island (the ‘northern’ haplotype group) split from the remaining little spotted kiwi samples. The partial DNA sequence obtained from sample AV17079 (collected near Napier, North Island; Table S2; Figure 1) was identical to the control region of haplotype A. The relationships between the three spotted kiwi lineages was largely unresolved, although there was support for great spotted kiwi and the northern haplotype group of little spotted kiwi forming sister lineages in the ML analyses (60% and 75% BS support for the expanded and reduced sample sets respectively). The two lineages of little spotted kiwi were not strongly supported as monophyletic in any of the analyses.

## Discussion

### Species Boundaries in Spotted Kiwi

The spotted kiwi sequences formed three strongly supported monophyletic clusters: great spotted kiwi, a ‘northern’ little spotted kiwi haplotype group comprising samples from northern North Island (Karamu, Waitomo and Napier) and a ‘southern’ little spotted kiwi haplotype group containing the remaining samples from the southern North Island and South Island. Although the exact relationships between these groups was unresolved in our analyses, the genetic distances between the ‘northern’ little spotted kiwi haplotype group, the ‘southern’ little spotted kiwi haplotype group and great spotted kiwi was similar to that previously used to delimit species in kiwi [2], [3]. However, using mitochondrial genetic distance alone to delimit species boundaries has long been criticized [4], [39], [40]. To date, morphological differences between bones of North Island and South Island little spotted kiwi have not been detected. Our results indicate considerable phylogenetic divergence between these populations, and suggest that a new morphological comparison is in order (*sensu* [41]). However, bones of the four larger kiwi species remain morphologically cryptic [6], so an absence of morphological differences between the bones of these little spotted kiwi may not reflect plumage differences or other isolating mechanisms. Additionally, obtaining longer mtDNA sequences and including nuclear DNA markers [23], [42] may contribute towards resolving relationships and determining taxon boundaries within spotted kiwi.

### Phylogeographic Patterns in Brown and Spotted Kiwi

The phylogenetic analyses clearly show differences in the levels and distribution of genetic variation in the two major morphological groups of kiwi (i.e. brown and spotted kiwi). Spotted kiwi exhibited fewer haplotypes overall than brown kiwi (10 versus 32, respectively, for the equivalent 871 bp dataset; Figure S1) and significantly lower haplotype and nucleotide diversities than brown kiwi species. In contrast to the widespread haplotype found in little spotted kiwi from the South Island (haplotype D), 18 haplotypes were detected from the equivalent 871 bp of DNA sequence in modern brown kiwi samples from the South Island, with a further 13 haplotypes found in the shorter ancient brown kiwi sequences (cytochrome *b* and control region only). The greater number of brown kiwi sequences available compared to those from spotted kiwi may partly account for the higher number of brown kiwi haplotypes. However, in contrast to the widespread haplotype D in little spotted kiwi, South Island brown kiwi haplotypes tended to be restricted to a single locality (Figure 1), with no haplotypes widespread and all three brown kiwi species had significantly higher haplotype and nucleotide diversities than the two spotted kiwi species.

Brown kiwi exhibited a higher level of variation in the South Island than in the North Island, whereas little spotted kiwi showed the opposite pattern. All three little spotted kiwi samples from the North Island that supplied full-length sequences possessed divergent haplotypes (haplotypes A, B and C). The origin of haplotypes A and B is perhaps a consequence of their location north of a marine barrier that transected the lower North Island until the last million or so years [43]. The sequence from the little spotted kiwi bone from Coonoor (haplotype C) was more closely related to South Island little spotted kiwi than to the other samples from the mainland of North Island. This result may mirror brown kiwi where ‘rowi’ type mitochondrial DNA extends across Cook Strait into the southern North Island [4] and is unsurprising given the recent formation of Cook Strait, perhaps only 0.5 Ma.

The high level of genetic structuring in brown kiwi has previously been attributed to their flightlessness, and thus presumed low levels of dispersal [2], [16]. However, little spotted kiwi, which are also flightless, lack a similarly high level of genetic structuring. There are a number of possible explanations for the differences in phylogeographic structure between brown and spotted kiwi. Firstly, they may differ in their dispersal behavior. Adults of all kiwi species are generally monogamous and remain in the same territory year round [12], [44]–[46]. However, juveniles and subadults of some species are known to disperse, although little data has been published. North Island brown kiwi juveniles have been recorded dispersing up to 22 km to find an unoccupied territory [47], [48]. In contrast, rowi juveniles do not disperse beyond the current population boundary and will fight adults for a territory [49]. There is also little published data available on whether there is any sex bias in dispersal. Male-biased sex dispersal can result in strong geographic structure in mitochondrial phylogenies, whereas female-biased dispersal leads to a lack of mitochondrial structuring [1]. North Island brown kiwi juvenile females have been reported as dispersing further than males, although sample sizes are small [48], [50] and this would result in a pattern of mtDNA structuring opposite to that actually observed [2], [3]. Even if more data on contemporary kiwi dispersal becomes available, it may not accurately represent the levels of dispersal that occurred prior to human arrival when kiwi populations were much larger and suitable habitat more continuous. Also, because little spotted kiwi are now restricted to islands or fenced sanctuaries, it may be difficult to use current observations to infer past dispersal behavior on the mainland.

Alternatively, the contrasting phylogeographic patterns of brown and spotted kiwi may suggest that the two kiwi groups responded differently to the Pleistocene glaciations. During the Last Glacial Maximum (LGM) temperatures were lower than present and accompanied by drought, strong winds and polar air masses causing severe frosts [51]. Much of the Southern Alps of the South Island were covered in ice during the glacial periods of the Pleistocene. Grasslands and shrublands with rare forest patches dominated most of the remaining areas of the South Island [52], although substantial forested refugia have been suggested for the north [52], [53] and south [54] of the South Island. In contrast, glaciation in the North Island is thought to have been much less severe with only small, localised areas of ice [55] and the survival of a large tract of continuous forest postulated for the north of the North Island [56], [57]. Genetic data has provided evidence of restriction of some New Zealand plants and animals to refugia during the LGM (e.g. *Metrosideros* trees, [57]; *Emeus* ratites, [43]; fungus beetles, [58]) and widespread survival of others (e.g. Hooker’s spleenwort fern, [59]; *Pseudopanax ferox* trees, [60]).

Under this scenario, South Island little spotted kiwi may have been restricted to one or more refugia during the LGM, thus reducing genetic diversity through a bottleneck effect. Little spotted kiwi could then have expanded out of the refugium following the end of the LGM to occupy their pre-human range. Conversely, if brown kiwi were not so restricted (occupying the areas of scrub and grassland present over much of the South Island at the time), they may have retained higher levels of genetic variation. However, contemporary studies indicate that North Island brown kiwi prefer to occupy mature forest rather than scrub [61]. Little spotted kiwi habitat preferences are largely unknown [62] although little difference in habitat use is seen on Kapiti Island where brown kiwi and little spotted kiwi overlap [62] and subfossil bones of these species frequently co-occur in deposits on the mainland (e.g., [63]).

There are several lines of evidence supporting different responses to climate cycling by brown and little spotted kiwi. Firstly, few little spotted kiwi bones of Holocene age have been recorded from sub-alpine areas and no little spotted kiwi bones are known from the LGM, suggesting that they were uncommon and may not have tolerated the environmental conditions present during that period. In contrast, subfossil ‘large’ kiwi bones (i.e. brown or great spotted kiwi) dating to the last glaciation have been found on both the east and west of the South Island [6], [15], [26], [63]. Secondly, brown kiwi, but not little spotted kiwi presently occur on Stewart Island (Figure 1), which was connected to the South Island during the last glaciation, but became isolated 12 000 yrs BP [64]. Little spotted kiwi bones have not been found on Stewart Island, although deposits of landbirds are not common [6], [65]. Collectively this evidence suggests that little spotted kiwi may not have been present in the south of the South Island during the last glaciation, which may account for the low haplotype diversity detected in the South Island. An alternative explanation is that all South Island little spotted kiwi may derive from dispersal from the North Island during the penultimate glacial, the last time when the North and South Islands were certainly connected [6].

It can be difficult to distinguish between alternative hypotheses when they generate similar mtDNA tree topologies [66]. Nuclear data could potentially determine whether the little spotted kiwi mtDNA phylogeny is a consequence of gender-biased dispersal and/or provide evidence for or against postglacial expansion. Additionally, accurate molecular dating has the potential to relate geographic structuring to Pleistocene glaciations. Two methods have previously been used to date divergences within kiwi. Firstly, a phylogenetic rate of 2% per million years was applied to cytochrome *b* data, resulting in species divergence estimates in the Pleistocene [2]. However, comparisons of avian mitochondrial clock rates using different calibrations (reviewed in [67]–[69]) indicate that there is considerable variation amongst rate estimates although most cluster around the 2% level. Secondly, a 25 million year old fossil from a distantly related ratite lineage was used to calibrate the kiwi phylogeny and so to date kiwi divergences to the late Miocene/early Pliocene [3]. These old divergence times were also used to suggest that an additional brown kiwi species, later described as rowi, be recognized because reproductive incompatibilities were assumed to have arisen during the inferred long period of isolation [3]. However, given the time dependency of molecular rates, using old calibration points to date more recent events is likely to lead to inaccurate date estimations [70], [71].

The most appropriate method for calculating divergence dates in kiwi is unclear. A kiwi-specific rate would be ideal but ratites, and indeed birds in general, suffer from a lack of suitable fossils to use in calibrating the molecular clock. A rate determined with ancient DNA from radiocarbon-dated kiwi samples (e.g., using the approach of [43]) is likely to be the most appropriate for examining recent divergences.

### The Recent History of Little Spotted Kiwi

The three South Island specimens collected in the last 60 years (Table S1) were confirmed as possessing little spotted kiwi mitochondrial DNA. Kiwi researcher Colin Roderick considered NMNZ OR.1174 & 23043 to be great spotted kiwi and this was reflected in government conservation agency reports of the time. For example, one of the reports states that there had been no reliable little spotted kiwi reports from the West Coast of the South Island ‘in the last forty years’ [72]. However, this was disputed at the time by researchers who believed these specimens are little spotted kiwi [15], [16].

The results presented here suggest that little spotted kiwi survived, and were widespread, on the mainland until quite recently. However, the possibility that the samples examined here were from hybrids cannot be discounted with the present genetic data (several hybrids between little spotted kiwi and rowi have previously been reported on the west coast of the South Island [73], [74], although in the one sample which has been DNA tested rowi contributed the mtDNA [73]). The decline of little spotted kiwi on the West Coast compared to rowi and great spotted kiwi may have increased the probability of their hybridization. Hybridization is more likely to occur where one parental species is rare and the other is common [75] and has been observed frequently in the declining New Zealand fauna [76].

Whether the little spotted kiwi on Kapiti Island, the current stronghold of this species, are natural or derive from a translocation has been the subject of much debate [19]. The limited sequence variation in the ‘southern’ haplotype group does not permit discrimination between hypotheses regarding the origin of the little spotted kiwi population on Kapiti Island. If this population was derived from a translocation by Europeans, then it was sourced from the South Island because very few little spotted kiwi were recorded from the North Island in historical times (there are only two historic records). Longer sequences, particularly of fast evolving DNA regions such as the control region, may help to identify variation within ancient little spotted kiwi from the South Island and therefore potentially discriminate between translocation hypotheses. Next-generation DNA sequencing technology provides a promising, and increasingly affordable, approach for producing large quantities of data from ancient samples such as these [77].

## Supporting Information

Figure S1 **Midpoint-rooted Bayesian phylogeny of the reduced sample set which included all spotted and brown kiwi samples and all loci.** Numbers above the branches represent posterior probablilities (PP), MP and ML bootstrap (BS) values, respectively. Only PP>0.70 and BS>50% are shown.
(TIF)

Table S1 **Details of ancient spotted kiwi samples used in this study.** The two GenBank number for cytochrome *b* correspond to non-contiguous fragments. Samples marked with an * were independently extracted at the University of Auckland. Museum abbreviations: CM - Canterbury Museum, NMNZ - Museum of New Zealand Te Papa Tongarewa, WO - Waitomo Caves Discovery Centre. NI =  North Island, SI =  South Island. The sequences from the three ancient great spotted kiwi specimens have been previously published [4].
(DOC)

Table S2 **Little spotted kiwi samples that either failed to amplify or only provided partial sequence.** Museum abbreviations: MNZ – Museum of New Zealand Te Papa Tongarewa, CM – Canterbury Museum, WO – Waitomo Caves Discovery Centre, AU – Auckland University Geology Department. NI =  North Island, SI =  South Island.
(DOC)

Table S3 **Details of modern spotted kiwi blood samples used in this study.** SI =  South Island.
(DOC)

Table S4 **Primer information.**
(DOC)
