# Supplementary figures and images for: Ancient DNA Analyses Reveal Contrasting Phylogeographic Patterns amongst Kiwi (Apteryx spp.) and a Recently Extinct Lineage of Spotted Kiwi
Source: PLoS One. 2012 Aug 2;7(8):e42384. doi: 10.1371/journal.pone.0042384 (PMC3410920; doi:10.1371/journal.pone.0042384)

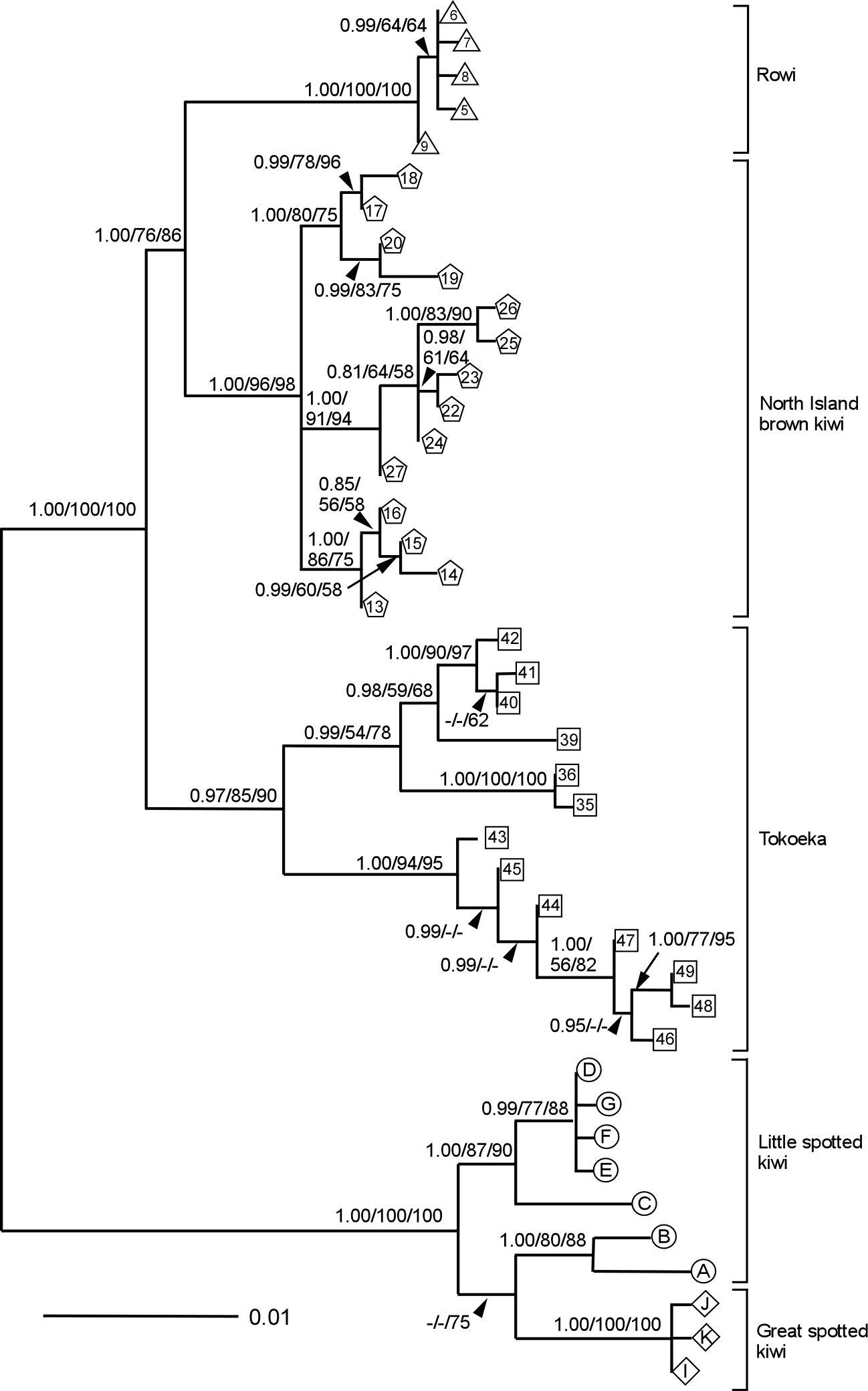

Supplement: Figure S1 — Midpoint-rooted Bayesian phylogeny of the reduced sample set which included all spotted and brown kiwi samples and all loci. Numbers above the branches represent posterior probablilities (PP), MP and ML bootstrap (BS) values, respectively. Only PP>0.70 and BS>50% are shown. (TIF) [file pone.0042384.s001.tif]
