# Supplementary material for: Ancient DNA Analyses Reveal Contrasting Phylogeographic Patterns amongst Kiwi (Apteryx spp.) and a Recently Extinct Lineage of Spotted Kiwi
Source: PLoS One. 2012 Aug 2;7(8):e42384. doi: 10.1371/journal.pone.0042384 (PMC3410920; doi:10.1371/journal.pone.0042384)
Supplement: Table S1 — Details of ancient spotted kiwi samples used in this study. The two GenBank number for cytochrome b correspond to non-contiguous fragments. Samples marked with an * were independently extracted at the University of Auckland. Museum abbreviations: CM - Canterbury Museum, NMNZ - Museum of New Zealand Te Papa Tongarewa, WO - Waitomo Caves Discovery Centre. NI = North Island, SI = South Island. The sequences from the three ancient great spotted kiwi specimens have been previously published [4]. (DOC) [file pone.0042384.s002.doc]

**Table S1.**  Details of ancient spotted kiwi samples used in this study. The two GenBank number for cytochrome *b* correspond to non-contiguous fragments. Samples marked with an * were independently extracted at the University of Auckland. Museum abbreviations: CM - Canterbury Museum, NMNZ - Museum of New Zealand Te Papa Tongarewa, WO - Waitomo Caves Discovery Centre. NI = North Island, SI = South Island. The sequences from the three ancient great spotted kiwi specimens have been previously published [4].

| Haplotype | Species | Museum no. | Museum | Locality | Sample type | Sample age (where known) | GenBank Accession no. (control region) | GenBank Accession no. (cytochrome *b*) | GenBank Accession no. (ATPase1) |
| --- | --- | --- | --- | --- | --- | --- | --- | --- | --- |
| A | *Apteryx owenii* | AV22817 | CM | Helectite Hole, Raglan, NI | Bone |  | FJ820096 | FJ820049, FJ820072 | JQ219178 |
| B | *Apteryx owenii* | WO255 | WO | Ann’s Cavern, Waitomo, NI | Bone |  | FJ820089 | FJ820042, FJ820065 | JQ219177 |
| C | *Apteryx owenii* | S.24478 | NMNZ | Coonoor, southern NI | Bone |  | FJ820092 | FJ820045, FJ820068 | JQ219176 |
| D | *Apteryx owenii* | OR.23036 | NMNZ | Banjo Creek, Westhaven Inlet, NW Nelson, SI | Skeleton | Collected 1978 | FJ820095 | FJ820048, FJ820071 | JQ219164 |
| E | *Apteryx owenii* | AV16713 | CM | Cave at Canaan, Takaka Hill, SI | Bone |  | FJ820090 | FJ820043, FJ820066 | JQ219168 |
| D | *Apteryx owenii* | S.27784.1  S.27784.2* | NMNZ | Earl Grey Cave, Takaka Hill, SI | Bone |  | FJ820094 | FJ820047, FJ820070 | JQ219162 |
| D | *Apteryx owenii* | S.001164 | NMNZ | Nelson | Toe pad | Collected 1914 | JQ219189 | JQ219195 | JQ219183 |
| D | *Apteryx owenii* | OR.1174 | NMNZ | Otututu or Rough River, Westland, SI | Toe pad | Collected 1952 | FJ820107 | FJ820061, FJ820084 | JQ277702 |
| G | *Apteryx owenii* | OR.23043 | NMNZ | Smyth River, South Westland, SI | Toe pad | Collected 1978 | FJ820091 | FJ820044, FJ820067 | JQ219163 |
| D | *Apteryx owenii* | OR. 22007* | NMNZ | Karangarua River, South Westland, SI | Feather/ Toe pad | Collected 1894 | FJ820093 | FJ820046, FJ820069 | JQ219174 |
| D | *Apteryx owenii* | OR.2069 | NMNZ | Lake Manapouri, SI | Toe pad | Collected between 1888-1896 | FJ820106 | FJ820060, FJ820083 | JQ219170 |
| D | *Apteryx owenii* | AV32392B | CM | Cave at Springhill, Southland, SI | Bone |  | FJ820099 | FJ820052, FJ820075 | JQ219169 |
| D | *Apteryx owenii* | DM6672 | NMNZ | Castle Rocks, Southland, SI | Bone | < 2100 BP^†^ | FJ820109 | FJ820063, FJ820086 | JQ219167 |
| D | *Apteryx owenii* | AV25301 | CM | King’s Cave, South Canterbury, SI | Bone |  | FJ820110 | FJ820064, FJ820087 | JQ219161 |
| F | *Apteryx owenii* | AV12648C | CM | Limestone fissure, Mt Somers Quarry, Canterbury, SI | Bone |  | FJ820098 | FJ820051, FJ820074 | JQ219173 |
| D | *Apteryx owenii* | S.33365 | NMNZ | Holocene Cave, Mt Cookson, SI | Bone | 2400-3000 BP^§^ | FJ820108 | FJ820062, FJ820085 | JQ219172 |
| D | *Apteryx owenii* | AV25141 | CM | West Coast, SI | Bone |  | FJ820097 | FJ820050, FJ820073 | JQ219171 |
| K | *Apteryx haastii* | S.23187 | NMNZ | Charleston, West Coast, SI | Bone |  | AY713331 | AY713364 | - |
| K | *Apteryx haastii* | AV19163 | CM | Charleston, West Coast, SI | Bone |  | AY713312 | AY713358 | - |
| H | *Apteryx haastii* | S.34491 | NMNZ | Mount Arthur, northern SI | Bone |  | AY713312 | AY713350 | - |

^†^Worthy 1998b

^§^Worthy & Holdaway 1995
