# Supplementary material for: Ancient DNA Analyses Reveal Contrasting Phylogeographic Patterns amongst Kiwi (Apteryx spp.) and a Recently Extinct Lineage of Spotted Kiwi
Source: PLoS One. 2012 Aug 2;7(8):e42384. doi: 10.1371/journal.pone.0042384 (PMC3410920; doi:10.1371/journal.pone.0042384)
Supplement: Table S2 — Little spotted kiwi samples that either failed to amplify or only provided partial sequence. Museum abbreviations: MNZ – Museum of New Zealand Te Papa Tongarewa, CM – Canterbury Museum, WO – Waitomo Caves Discovery Centre, AU – Auckland University Geology Department. NI = North Island, SI = South Island. (DOC) [file pone.0042384.s003.doc]

**Table S2.** Little spotted kiwi samples that either failed to amplify or only provided partial sequence. Museum abbreviations: MNZ – Museum of New Zealand Te Papa Tongarewa, CM – Canterbury Museum, WO – Waitomo Caves Discovery Centre, AU – Auckland University Geology Department. NI = North Island, SI = South Island.

| Museum Number | Museum | Locality | Sample type | GenBank Accession nos. |
| --- | --- | --- | --- | --- |
| DM6663 | NMNZ | Castle Rocks, Southland, SI | bone | FJ820113, FJ820088 |
| OR.019281 | NMNZ | Poukawa, NI | bone | - |
| OR.009651 | NMNZ | Poukawa, NI | bone | - |
| OR.244781 | NMNZ | Coonoor, NI | bone | - |
| AV17079 | CM | Cave 30 miles from Napier, NI | bone | FJ820112 |
| AV2852 | CM | East Coast, SI | bone | - |
| AV19575 | CM | Te Kuiti, Waitomo, NI | bone | - |
| AV23470 | CM | Mahoenui, Waitomo, NI | bone | - |
| AV15060 | CM | Pyramid Valley, Canterbury, SI | bone | - |
| AV22701 | CM | Tom Bowling Bay, Northland, NI | bone | - |
| AV32392A | CM | Springhill, Southland, SI | bone | FJ820111 |
| AV23067A | CM | Kings Cave, Canterbury, SI | bone | - |
| AV23067B | CM | Kings Cave, Canterbury, SI | bone | - |
| W0270.7 | WO | Junior Mudball Cave, Waitomo, NI | bone | - |
| AU4073.60 (N02/f047) | AU | Tom Bowling Bay, Northland, NI | bone | - |
| AU4935.2 (N02/f049) | AU | Tom Bowling Bay, Northland, NI | bone | - |
| AU4712.9 (N02/f055) | AU | Whareana Beach, Northland | bone | - |
| AU4712.10 (N02/f055) | AU | Whareana Beach, Northland | bone | - |
| AU4917.1 (N02/f055) | AU | Whareana Beach, Northland | bone | - |
| AU4917.3 (N02/f055) | AU | Whareana Beach, Northland | bone | - |
