# Supplementary material for: Ancient DNA Analyses Reveal Contrasting Phylogeographic Patterns amongst Kiwi (Apteryx spp.) and a Recently Extinct Lineage of Spotted Kiwi
Source: PLoS One. 2012 Aug 2;7(8):e42384. doi: 10.1371/journal.pone.0042384 (PMC3410920; doi:10.1371/journal.pone.0042384)
Supplement: Table S3 — Details of modern spotted kiwi blood samples used in this study. SI = South Island. (DOC) [file pone.0042384.s004.doc]

**Table S3.** Details of modern spotted kiwi blood samples used in this study. SI = South Island.

| Species | Haplotype | National Tissue Collection code | Provenance | GenBank accession no. (control region) | GenBank accession no. (cytochrome *b*) | GenBank accession no. (ATPase1) |
| --- | --- | --- | --- | --- | --- | --- |
| *Apteryx owenii* | D | CD899 | Kapiti Island | FJ820115 | FJ820081, FJ820058 | JQ219165 |
|  | D | CD1206 | Kapiti Island | FJ820104 | FJ820082, FJ820059 | JQ219166 |
|  | E | WS1764 | D’Urville Island | FJ820105 | FJ820078, FJ820055 | JQ219175 |
| *Apteryx haastii* | I | GS14 | Ugly River, NW Nelson, SI | FJ820114 | FJ820080, FJ820057 | JQ219182 |
|  | J | M3 | Heaphy, NW Nelson, SI | FJ820101 | FJ820076, FJ820053 | JQ219179 |
|  | J | FT2921 | Kahurangi, NW Nelson, SI | FJ820103 | FJ820079, FJ820056 | JQ219181 |
|  | J | FT2920 | Kahurangi, NW Nelson, SI | JQ219192 | JQ219196 | JQ219187 |
|  | K | GS10 | Deception River, Arthurs Pass, SI | JQ219191 | JQ219200 | JQ219186 |
|  | K | GS11 | Deception River, Arthurs Pass, SI | JQ219190 | JQ219199 | JQ219185 |
|  | J | FT2922 | Big River, Lewis Pass, SI | JQ219193 | JQ219197 | JQ219188 |
|  | J | GS17 | Ugly River, NW Nelson, SI | JQ219194 | JQ219198 | JQ219184 |
|  | K | GS21 | Taramakau River, Arthurs Pass, SI | FJ820102 | FJ820077, FJ820054 | JQ219180 |
