# Supplementary material for: Ancient DNA Analyses Reveal Contrasting Phylogeographic Patterns amongst Kiwi (Apteryx spp.) and a Recently Extinct Lineage of Spotted Kiwi
Source: PLoS One. 2012 Aug 2;7(8):e42384. doi: 10.1371/journal.pone.0042384 (PMC3410920; doi:10.1371/journal.pone.0042384)
Supplement: Table S4 — Primer information. (DOC) [file pone.0042384.s005.doc]

**Table S4.** Primer information

| Primer | Sequence (3’ to 5’) | Product size (bp*) |
| --- | --- | --- |
| Control region | | |
| Kcf2 with  Kcr2 | ccttgtaggcaaatacagt | 190 |
|  | gtgttgaatcaggaaatcc |  |
| Cytochrome *b* | | |
| Kcytb1** with  LSKcytA | AAACATCTCCGCGTGATGAAACTTCGGAT | 191 |
|  | GATGCTCCGTTTGCATGTAG |  |
| LSKcytB with  LSKcytb2 | ATCCATCGCCCATATCTGTC | 246 |
|  | AACTGTAGCCCCCCAAAATGATATTTGTCCCCA |  |
| LSKcytD with  LSKcytE | TCCCATACATCGGACAAACC | 214 |
|  | GTATGGGTGGAAGGGGATTT |  |
| ATPase | | |
| ATPase1F with  ATPase1R | AACTCAACCCAAACCCATGA | 257 |
|  | GGATGGGAGCAATAAAGCAG |  |

* bp: base pairs excluding primers, ** Primer from Shepherd and Lambert 2008
